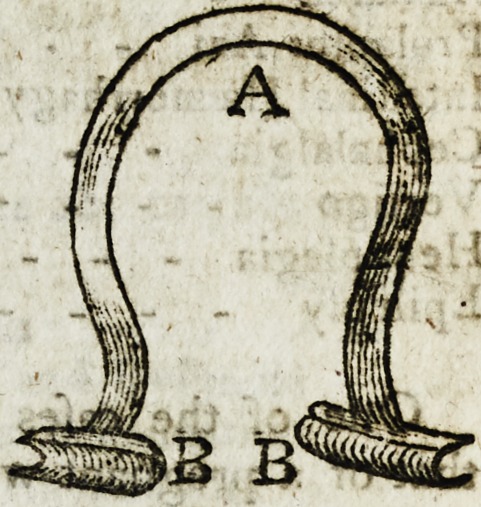# To the Editors of the Medical and Physical Journal

**Published:** 1800-08

**Authors:** Thomas Richmond

**Affiliations:** Paisley


### *To the Editors of the Medical and Phyſical Journal.*

GENTLEMEN,

PERMIT me to offer the model of a Speculum Oculi, for inſertion in the Medical and Phyſical Journal. It conſiſts of two parallel grooves, connected by a gently elaſtic ſteel arch; when we wiſh to apply it, the eye-lids are firſt opened, till they are inſerted in the grooves of the Speculum; the ſpring is then let go, and the eye is expoſed by the action of the ſpring, which is held in its place by the re-action of the eye-lids, without the aid of an aſſiſtant; an improvement, deſirable in the operation of extracting cataract.

The anecdote which gave riſe to the idea of the inſtrument, was as follows: A late medical gentleman in Edinburgh, performing the operation of extraction, employed the uſual ſpeculi of drawing down the under eye-lid by the hook, and elevating the upper one by the curved wire and an aſſiſtant. His aſſiſtant was not ſufficiently ſkilful or attentive, ſo that when the cornea was cut through, and the extraction about to be performed, an unguarded preſſure was made upon the eye with the wire, and the whole humours burſt out. It is evident that this inſtrument will prevent the like accident in future. I have tried it in real practice, and it has completely anſwered my expectations, as well as my other medical friends. I remain,

GENTLEMEN,

Your moſt obedient,

*Paiſley, July 8, 1800.* THOMAS RICHMOND.

A. The ſteel arch. B. B. The parallel grooves.

## Figures and Tables

**Figure f1:**